# Draft Genome Sequences of 10 Pseudomonas sp. Isolates from the Active Layer of Permafrost in Ny Ålesund, Svalbard, Norway

**DOI:** 10.1128/mra.00201-22

**Published:** 2022-05-16

**Authors:** Katie Sipes, Raegan Paul, Tullis C. Onstott, Tatiana A. Vishnivetskaya, Karen G. Lloyd

**Affiliations:** a Microbiology Department, University of Tennessee, Knoxville, Tennessee, USA; b Center for Environmental Biotechnology, University of Tennessee, Knoxville, Tennessee, USA; c Department of Environmental Science, Aarhus University, Roskilde, Denmark; d Department of Geosciences, Princeton University, Princeton, New Jersey, USA; University of Arizona

## Abstract

Ten distinct isolates from the genus Pseudomonas were isolated in culture. The genomes of these isolates were sequenced using the Illumina MiSeq platform and assembled in order to provide insight into the metabolic and carbon-degrading potential of bacteria residing in soils at high latitudes.

## ANNOUNCEMENT

Pseudomonas is a genus of gammaproteobacteria that is found in many environments. Members of the genus Pseudomonas are known for their ability to adapt to new environmental conditions, their metabolic versatility, and their ability to utilize different compounds as a source of carbon, nitrogen or phosphorus, which makes them ubiquitous. Pseudomonas sp. strains have been isolated from active-layer sediment of permafrost (1). As more microorganisms are cultivated from permafrost-affected environments, we can gain a deeper understanding of the community structure and predict microbial responses to climate change.

In this report, draft genome sequences were obtained for 10 Pseudomonas sp. isolates from the active layer of permafrost in Ny Ålesund, Svalbard (78°55.237′N, 011°50.495′E). The sediment samples were collected from two cores, BPF1 (0 to 58 cm) and BPF2 (0 to 30 cm), unearthed using a SIPRE auger drill from fully frozen ground in April 2018. Both cores were trimmed and separated into intervals of 2-cm vertical depths following an aseptic procedure. The sediment was mixed with sterile phosphate-buffered saline (PBS) at a ratio of 1:1. The organisms were then grown on Reasoner’s 2A (R2A) agar plates. The plates were incubated at 4°C for 3 weeks. Once the bacteria grew into distinct colonies, they were transferred to 10 mL R2A broth medium. The bacteria were then grown at 4°C and stationary conditions for 3 weeks. After growth, the cells were pelleted by centrifugation at 5,000 × *g* for 5 min, then resuspended in a buffer from the Qiagen DNeasy PowerSoil kit (Qiagen, Germany). This kit was used to extract genomic DNA from each isolate. Following extraction, the DNA was prepped using a Nextera XT library prep kit and sequenced using an Illumina MiSeq instrument with v3 chemistry (600 cycles, 2 × 300-bp format) at the University of Tennessee, Knoxville Center for Environmental Biotechnology (Table 1). The genomic data were retrieved from Illumina BaseSpace and assembled using SPAdes v3.13.0 (2) at https://www.kbase.us/ using default parameters (see link in “Data availability”) (3). The quality of the assemblies was reviewed using QUAST v4.4 (4). Annotations were performed using Prokka v1.14.6 (5).

**TABLE 1:** Strain information, average nucleotide identity results, and sequencing metrics for the whole-genome sequences in this study

Strain name	Svalbard site origin (depth [cm])	Closest match^*a*^	Identity (%)	GC content (%)	Total length (bp)	Avg read length (bp)	No. of reads	No. of contigs	*N*_50_ (bp)
B3	BPF1 (24–36)	Pseudomonas silesiensis strain ILQ215	99	58.62	11,789,631	254.4	2,487,662	2,001	14,511
B4	BPF1 (48–58)	Pseudomonas mandelii strain JZY4-67	99	58.75	6,458,228	259.11	3,314,326	147	146,853
B5	BPF1 (0–12)	Pseudomonas sp. strain PF1B2	100	58.75	6,495,411	260.27	3,829,288	210	125,761
B7	BPF2 (20–30)	Pseudomonas sp. strain PF1B2	100	57.93	12,755,968	258.21	2,947,652	2,088	47,400
E5	BPF1 (36–48)	Pseudomonas sp. strain PAMC 27331	100	59.03	9,163,259	259.98	2,493,944	358	30,476
E6	BPF1 (0–12)	Pseudomonas mandelii strain UTB_118	99	59.31	5,205,891	259.78	3,091,498	147	43,242
E7	BPF2 (20–30)	Pseudomonas mandelii	100	59.12	11,138,125	260.95	2,900,796	277	52,862
G16	BPF1 (48–58)	Pseudomonas mandelii strain UTB_115	99	62.87	10,219,356	255.91	3,646,716	74	269,342
G17	BPF1 (48–58)	Pseudomonas sp. strain PAMC 27357	99	61.77	8,423,581	243.1	12,297,290	51	281,547
G19	BPF1 (0–12)	Pseudomonas sp. strain PF3B13	99	52.59	9,857,464	258.45	4,733,580	56	182,733

a Based on a search of NCBI’s 16S rRNA gene database.

In this article, we report the isolation and genome sequencing of 10 Pseudomonas strains, namely, strains B3, B4, B5, E5, E6, G16, G17, and G19 (from the BPF1 active-layer core) and strains E7 and B7 (from BPF2). The species of each bacterial strain was determined by comparison of its 16S rRNA gene to the NCBI database (Table 1). The Pseudomonas sp. isolates were analyzed for their carbon-degrading activity, and their genomes were analyzed for the presence of catabolic genes corresponding to those activities (6). Out of seven enzymes tested, all isolates had the highest activity of leucine aminopeptidase and the highest number of peptidase genes (6). The data presented in this article extend our previous knowledge on the microbial diversity of organisms present in the active layer of permafrost-affected soil.

### Data availability.

The whole-genome sequences and SRA submissions can be found at NCBI GenBank under BioProject accession number PRJNA649544. The workflow of the metagenomic analysis can be found at the following permanent link with a free account: https://www.kbase.us/.
